# Exposure to Aflatoxin M1 through Milk Consumption in Tehran Population, Iran

**DOI:** 10.22037/ijpr.2019.1100764

**Published:** 2019

**Authors:** Hamid Khaneghahi Abyaneh, Alireza Bahonar, Negin Noori, Hassan Yazdanpanah, Mohammad Hossein Shojaee AliAbadi

**Affiliations:** a *Department of Food Hygiene and Quality control, Faculty of Veterinary Medicine, University of Tehran, Tehran, IR Iran.*; b *Food Safety Research Center, Shahid Beheshti University of Medical Sciences, Tehran, IR Iran.*; c *Toxicology and Pharmacology Department, School of Pharmacy, Shahid Beheshti University of Medical Sciences, Tehran, IR Iran. *; d *Toxicology Department, Faroogh Life Sciences Research Laboratory, Tehran, IR Iran.*

**Keywords:** Aflatoxin M_1_, Milk, Exposure assessment, Tehran, Iran

## Abstract

Milk would be contaminated with Aflatoxin M1 (AFM_1_), if it was obtained from lactating animal which fed with feedstuffs containing Aflatoxin B1 (AFB_1_). AFM_1_ is classified as group 2B**, **possibly carcinogenic to humans and its exposure to AFM_1_ through milk consumption is a public concern. The purpose of this study was to determine the AFM_1_ exposure through liquid milk consumption for adult consumers in Tehran. Forty-five samples including raw, pasteurized, and UHT milk samples were collected from markets in different cities of Tehran province in January and February 2017. The AFM_1_ was determined by HPLC method after immunoaffinity column clean up. Also, the milk intake was calculated using household budget survey. Finally, the daily intake of AFM_1_ through milk consumption was estimated using a deterministic approach. From total 45 samples, AFM_1_ was detected in 36 (80%) samples, although none of the analyzed samples were exceeded Iran legal limit of 0.1 µg/kg. On the basis of the average milk intake, the mean daily exposure to AFM_1_ was estimated between 0.03 ng/ Kg BW per day (lower bound estimate) and 0.06 ng/ Kg BW per day (upper bound estimate) and the 95th percentile daily exposure was calculated at 0.14 ng/ Kg BW per day. According to these values, it should be expected that the adults of Tehran population are not exposed to a significant risk of Hepatocarcinoma associated with AFM_1_ intake through milk consumption.

## Introduction

Aflatoxin M_1_ (AFM_1_) is a monohydroxylated metabolite of aflatoxin B_1_ (AFB_1_) (1, 2). Feed may be contaminated with AFB_1_ where the environmental conditions are favorable for mold growth, and when AFB_1_ contaminated feed consume by dairy ruminant animals, AFB_1_ is transformed to AFM_1_ by means of microsomal cytochrome P450-associated enzymes in liver and excreted in milk at a rate of 0.3-6.2 percent of ingested AFB_1_, depending on the AFB_1_ amount of feed (2–4).

AFM_1_ contamination of milk and dairy products has been reported from many countries (5–8), most of which are located in the Mediterranean and the Middle East region, where environmental conditions is suitable for mold growth in agricultural products used as animal feed (6). It is noticeable that the presence of AFM_1_ in milk and dairy products were reported from Iran in many published articles during the last decade (7–9). Furthermore, some studies from Iran have demonstrated high frequency rate of AFM_1_ contamination in milk and dairy products (7).

In terms of food safety, the importance of AFM_1_ is due to its carcinogenic potency (1, 5). The International Agency for Research on Cancer (IARC) categorized AFM_1 _as a Group 2B human carcinogen in 1993 (1, 10). Therefore, the Joint FAO/WHO Expert Committee on Food Additives (JECFA) has not established a maximum tolerable daily intake (TDI) for AFM_1_, because AFM_1 _intake levels even less than 1 ng/kg body weight (BW) per day increase the risk of liver cancer, so recommended AFM_1 _level of milk and dairy products should be as low as reasonably achievable (ALARA) (7, 11). The greatest potential of milk for introducing AFM_1_ into the human diet has been demonstrated, so milk and dairy products consumption contribute significantly for the human exposure to AFM_1_ which is a serious public concern (Ruangwises & Ruangwises, 2010). The common AFM_1_ exposure assessment plan is based on the combination of the AFM_1_ occurrence data with milk intake data using a deterministic approach (12). However, the AFM_1 _levels in milk is usually low, and only long-term intake of such low levels is associated with the occurrence of diseases such as Hepatocarcinoma in humans (1). So, the main health concern is relevant to areas where have high milk consumption per capita, as well as children and adolescents who have the higher proportion of milk intake per kg of body weight. As a result, considering food consumption pattern and economic considerations (8), the legal maximum limits for AFM_1_ are different in various countries (2, 8, 13, 14). 

Several methods have been developed for measuring AFM_1_ in milk such as ELISA as a screening method, and high performance liquid chromatographic techniques (HPLC) after immunoaffinity clean-up as a confirmatory method (5). 

Several studies have reported the exposure to AFM_1_ through milk and dairy products consumption from different countries in worldwide (11, 12, 15–18). At the international level, AFM_1_ daily intake through milk consumption by European Union, Latin America, Far Eastern, Middle Eastern and African population was respectively estimated as 0.11, 0.058, 0.20, 0.10, and 0.002 ng/kg BW per day, within the framework of GEMS/Food regional diets (5, 19)

In Iran, AFM_1_ contamination of varied portion of analyzed milk samples was reported in almost all published studies so far (20) and based on the obtained results, occurrence and levels of AFM_1_ contamination seem to be a public health concern in winter season and humid climate regions of Iran, particularly for children (2, 21). However, AFM_1_ exposure through milk and traditional dairy product consumption in an adult population of four west (Kermanshah, Ilam, Hamadan, and Kurdistan) provinces is only one published report regarding AFM_1_ intake from Iran (21). 

The purpose of this study was to determine the exposure to AFM_1_ through raw, pasteurized and UHT milk consumption in an adult urban population of Tehran province in Iran.

## Experimental

*Sampling*

A total of 45 milk samples, including 25 raw and 20 Heat-treated milk (including 16 Pasteurized and 4 UHT milk) the samples were obtained from markets in different cities of Tehran province, during January and February 2017.

Raw milk samples were collected with sampler jars directly from milk-holding tanks in the traditional dairy product markets. After stirring the milk-holding tank, the equal amount of milk was collected from each tank in a market, and then pooled together, and finally, 500 mL milk sample was transferred to a disposable pet container.

Pasteurized and UHT milk samples were obtained from different supermarkets or hypermarkets in original packaging. Only one packaging was selected from each available brand. Then, 500 mL milk sample from each pack was transferred as a sample to a disposable pet container. Soon after collection, the samples were transported to the laboratory in an icebox with ice packets, and then stored at -20 °C and protected against light until further analysis for AFM_1_.

*Apparatus, chemicals and reagents*

Agilent Technologies 1200 Series HPLC system (USA) consisted of binary pumps and a fluorescence detector was used to determine AFM_1_ and equipped with a custom built oven column. 

Separation was achieved using an Agilent Eclipse XDB- C18 column (4.6 × 150 mm, 5 μm). 

Immunoaffinity column obtained from Libios (PuriFast Afla, Libios, France).

Chemicals and reagents were HPLC grade including: cetonitrile (Merck, Germany), Methanol (Merck, Germany), Deionized Water (Heal Force, China), Sodium Chloride (Merck, Germany), Potassium Chloride (Merck, Germany), Potassium Dihydrogen Phosphate (Aldrich, Germany), Disodium Hydrogen Phosphate (Carlo Erba, Italy), Nitric Acid 65% (Merck, Germany), and Potassium Bromide (Merck, Germany).

AFM_1_ stock standard solution was prepared from Sigma Chemical Co. (Sigma, USA) and kept frozen at −20 °C prior to the experiment. Working standard solutions AFM_1_ at concentrations of 0.25, 0.50, 0.75, 1.00, 1.25, and 1.50 μg/L in mobile phase were used to obtain the calibration curve.

*Extraction* *and clean up procedure*

According to the official national standard of ISIRI, No. 7133 based on ISO 14501/IDF 171 (22), the frozen milk samples were thawed using a water bath at 35 °C to 37 °C, and then liquid milk was centrifuged at 4500 × g for 15 minutes and upper fat layer discarded completely. 

The skimmed milk was filtered through a paper filter (GVS Filter Technologies; Italy) and then 50 mL of it was passed through immunoaffinity column at flow rate of 1 mL/min. 

Immunoaffinity column was previously brought to the room temperature by passing 10 ml of Phosphate buffered saline (PBS)]. Next, 15 mL of PBS was used for washing sample container and then passed through immunoaffinity column. The column was washed with a mixture of acetonitrile and methanol (3:2 v/v), twice (each time with 500 μL). 

The eluate was collected in a conical tube and evaporated to dryness using a gentle stream of nitrogen. The residue was dissolved in 1 mL of mobile phase and then a 200μL aliquot was injected into LC system and filtered through a syringe filter (0.2 µm PTFE; USA).

*Quantitative* *analysis by HPLC*

The HPLC conditions for quantitative analysis of AFM_1_ were as follows: column temperature 40 °C and mobile phase consisted of water: methanol: acetonitrile (60:30:10 v/v) + 350 µL HNO3 4M + 120 mg/L KBr pumped at a flow rate of 1 mL/min. Excitation and emission wavelengths of fluorescence detector were 362 and 435 nm, respectively. The retention time for AFM_1_ was 5.8 min.

For identification of AFM_1_ peak in the sample chromatogram, its retention time was compared with that of the analyzed AFM_1_ standard under the same conditions. Using the equation of calibration curve, the area under the curve of sample chromatogram was calculated for quantitation of AFM_1_. The limits of detection (LOD) and quantitation (LOQ) of the current method were 0.01 and 0.03 μg/L, respectively. 

*Statistical Analysis*

Mean, standard deviation (SD), and 95 percentile of AFM_1_ concentration in milk samples were statistically analyzed by the Data Analysis tools of Microsoft Excel 2010 for data analysis. 

*Calculation of exposure*

In this study, daily intake of AFM_1_ was calculated using the deterministic approach explained by International Program on Chemical Safety (IPCS) (23).

Based on Global Environment Monitoring System (GEMS)/Food guidelines, as the proportion of censored data (results reported below LOD and/or LOQ) exceeded 60%, two scenarios were adopted and used for calculation purposes: 

(1) the upper bound of mean (UB) computes after replacing the LOQ instead of the results that were lower than LOQ and LOD instead of the results that were lower than LOD; (2) the lower bound of mean (LB) computes by replacing the LOD instead of the results were lower than LOQ and zero instead of the results , lower than LOD (23–25).

The milk consumption per capita in Iran was calculated using the average milk consumption by a household from March 2016 to February 2017 as provided by the Household Budget Survey in Urban Areas of Iran in 2017 divided by the average size of urban households in this year (26). Then, milk consumption per capita by urban population of Tehran province was estimated by comparison whole country with Tehran household expenditure for purchasing milk types (27). 

Taking into account the variability that exists in food consumption patterns within our studied population, the milk consumption per capita was calculated using coefficients obtained from the study performed by Nasimi *et al*. They demonstrated per capita milk consumption of two top income decile is almost three times more than two lowest income decile (28). Moreover, the pattern of household milk consumption is assumed to be raw milk or heat-treated milk or both.

Eventually, the mean and 95 percentile exposure levels (p95) to AFM_1_ were calculated by combining the mean and percentile 95 of the AFM_1_ concentrations with the milk intake using the following formula: 

Daily intake [ng/kg BW /day] =[AFM1 concentration (ng/kg )][Daily milk consumption (Kg/day)][body weight BWkg ]

## Results

*Method performance*

Each day a set of working standard solutions were injected to construct the calibration curve. The accepted linearity of the calibration of minimum R^2^> 0.98 was obtained at the working range. For quality control, recovery test was performed by spiking of the blank milk samples with known amounts of AFM_1_ (0.1 μg/L). Mean recovery rates and relative standard deviations were 90.6 ± 5.7%.

**Figure 1:**
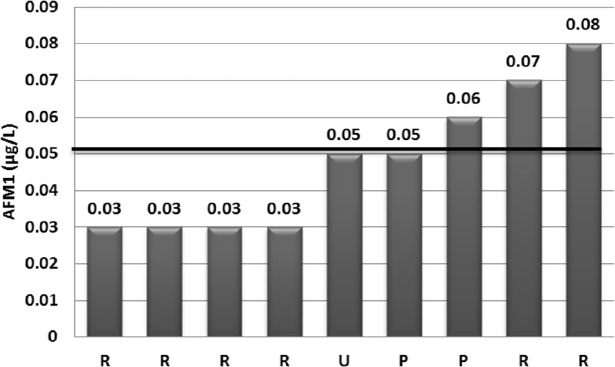
Distribution of AFM1 concentration of the analyzed milk samples above than the limit of quantification value (0.03 µg/kg). The mean of AFM1 concentration for these samples (0.047 µg/L) is represented with a horizontal line (R: raw milk, P: pasteurized milk, U: UHT milk)

**Table 1:** AFM1 contamination in different types of milk samples collected from markets of Tehran province during January and February, 2017

**Descriptive data**		**Raw milk**	**Heat-treated milk ** ***a***
Frequency rate (%)		18/25 (72)	18/20 (90)
Mean (± SD) μg /kg	*Positive samples*	0.045 **(**0.234)	0.0533 (0.0057)
	*Total samples * *b*	0.0155- 0.0315	0.0156-0.0280
Percentile 95	*Positive samples*	0.075	0.059
	*Total samples*	0.062	0.050

*a*: Including Pasteurized milk and UHT milk.

*b*: *Upper limit was *Calculated by replacing the results below LOQ by LOQ and results below LOD by LOD, *Lower limit was *calculated by replacing the results below LOQ by LOD and results below LOD by zero.

**Table 2:** AFM1 intake through milk consumption by each urban household’s member of Tehran province

**Milk type**	**Milk consumption pattern**		**AFM**_1_ ** intake** **(ng/ person per day)**			**AFM**_1_ ** intake ** ^g^ **(ng/ Kg BW per day)**		
	Consumer group	Milk intake	Mean		Percentile (P95)^ f^	Mean		Percentile (P95)
		(g/day) ^c^	LB ^d^	UB ^e^		LB	UB	
Raw milk	*High consumer*	222	3.44	6.99	13.21	0.05	0.10	0.19
	*Moderate consumer*	148	2.29	4.66	8.86	0.03	0.07	0.13
	*Low consumer*	74	1.15	2.33	4.40	0.02	0.03	0.06
Heat-treated milk ^a^	*High consumer*	222	3.46	6.22	17.09	0.05	0.09	0.24
	*Moderate consumer*	148	2.31	4.14	11.40	0.03	0.06	0.16
	*Low consumer*	74	1.15	2.07	5.70	0.02	0.03	0.08
Liquid milk ^b^	*High consumer*	222	3.46	6.56	14.87	0.05	0.09	0.21
	*Moderate consumer*	148	2.30	4.37	9.92	0.03	0.06	0.14
	*Low consumer*	74	1.15	2.19	4.96	0.02	0.03	0.07

* a*: Including Pasteurized and UHT milk; *b*: Including Raw, Pasteurized and UHT milk; *c*: The pattern of household milk consumption is assumed to be only raw milk or only heat-treated

milk or both; *d*: LB: Lower bound estimate; *e*: UB: Upper bound estimate; *f*: P95: 95 Percentile; *g*: The average body weight for adults is assumed 70 kg;.

*Occurrence of AFM1 in milk*

AFM_1_ was detected in 36 (80%) from 45 analyzed milk samples. However, the AFM_1_ level was 0.03µg/kg (LOQ) or higher in 9 (20%) samples. The concentration of AFM_1_ in 12 from 25 raw milk (48%) and 15 from 20 (75%) heat-treated milk positive samples were lower than LOQ. Distribution of AFM_1_ contamination was presented in Figure 1. As shown in this Figure, AFM_1_ concentration in 3 (6.66%) of the milk samples exceeded the EU maximum tolerance limit for AFM_1_ (0.05 µg/kg), although none of the analyzed samples were exceeded Iranian legal limit (0.1 µg/kg) (14) and the Codex Alimentarius criterion of 0.5 μg/kg (8, 13). The upper and lower limit of mean AFM_1_ concentrations was 0.016 and 0.030 μg/kg and its 95th percentile was 0.667 μg/kg, whereas mean (± SD) of AFM_1_ levels for the positive samples was 0.048 ± 0.019 μg/kg. The descriptive data of AFM_1_ contamination occurrence by type of sample was presented in Table 1.

*AFM*_1_ *intake estimate*

The milk consumption per capita by urban population of Tehran province was calculated 27, 54, and 81 kg/year (74, 148 and 222 gr/day) for population groups with high, moderate, and low milk consumption, respectively. 

The average body weight for adults was assumed 70 kg. Accordingly, the mean daily exposure to AFM_1_ was calculated with the range between 0.03 (lower bound estimate) and 0.06 (upper bound estimate) ng/Kg BW per day for each member of the urban households in Tehran province. 

The Mean and 95 percentile (p95) exposure to AFM_1_ through raw and heat-treated milk consumption were presented in Table 2.

## Discussion

Milk and dairy products are an important part of the human diet, notably for infants and children, due to its richness in certain nutrients such as protein, calcium, riboflavin, phosphorus, potassium, vitamins A and D, and its usability at all ages (29). However, they could contain some contaminants such as AFM_1_(9) and AFM_1_ intake through milk consumption is an important health concern because of AFM_1_ carcinogenic properties, especially as there are no preventing procedures for the complete elimination of AFB_1_ in feeds, as well as suitable weather conditions for the growth of fungi and production of mycotoxins in feed. On the other hand, the resistance of AFM_1_ to the heat treatment and mild acidic conditions used in dairy processing were demonstrated (30, 31). So, the dairy products are contaminated with AFM_1_ if raw milk used for processing is contaminated with AFM_1_ (32, 33). The mixing of bulk milk consignments of different contamination levels is the only process currently applied (34).

Our results were lower than AFM_1_ exposure level reported from four west provinces of Iran (0.242 ng/kg BW per day) (21), Sao Paulo, Brazil (0.18 and 0.14 ng/kg BW per day) (15, 16), Catalania region, Spain (0.036 and 0.043 ng/ person per day for male and female, respectively) (12), and Serbia (1.420, 0.769 and 0.503 ng/kg BW per day during February, April and May 2013) (11) and higher than AFM_1_ exposure level reported from France (0.01 ng/kg BW per day) (17), while being agreement with a report from Rabat, Morocco (3.26 ng/person per day) (18). Level of AFM_1_ contamination and milk intake per capita are the most important factors affecting the exposure reported in these studies.

The estimated mean of AFM_1_ concentration in the present study was higher than the previous values ​​reported from Iran by Sheikhloie and Safarpour (35) and Nowrozi and Kazemi (36), while being lower than the AFM_1_ average reported from Iran by kamkar *et al*. (37), Rezaei *et al*. (38) and mashak *et al*. (39). However, the reported results by Movassaghghazani and Ghorbiani (40) and Sohrabi and Gharahkoli (41) from Iran were similar to our results. Season of sampling and climate condition of the study area; number and type of analyzed samples, used method for AFM_1_ analysis were the factors influencing the AFM_1_ concentration reported by different researchers. It is remarkable, all samples in our study were collected in the winter season and, as reported by some researchers, the AFM_1_ contamination of raw milk in this season is significantly at higher level compared with other seasons. This is because of the lactating animals are fed with greater amounts of silage and concentrate feeds in cold seasons which may be contaminated with higher levels of AFB_1_ (21, 42, 43). However, the occurrence of AFM_1_ in samples collected from a modern dairy farm in winter season is higher than summer season versus traditional farm (43).

The lower consumption of milk in Iran than the recommended daily intake by optimal food basket (44) was another reason for being low AFM_1_ exposure that was obtained in our study. However, AFM_1_ intake in high consumers was up to three times more than low consumers, because of the milk and dairy products expending are strongly dependent on household income (28). Meanwhile, AFM_1_ exposure in children and adolescents who have more proportion of milk intake per kg of body weight are higher than adults. Moreover, AFM_1_ exposure in Tehran population will increase in the long term, because of an expected augment in milk consumption to the recommended daily intake by enhancing household livelihood and public knowledge.

## Conclusion

This study represents one of the first insights into the AFM_1_ exposure through milk consumption in Iran population. Although the levels of AFM_1_ contamination in our collected milk samples and per capita milk consumption of our study population and so estimated AFM_1_ intake in an adult of Tehran population were low, the contribution of such low levels of AFM_1_ intake in increasing the risk of hepatocarcinoma could not be ignored, notably regarding an expected increase in milk consumption to the recommended daily intake in the long term. Therefore, systematic AFM_1_ monitoring program in raw milk should be performed in along the time. Moreover, as an important strategy to protect consumers against AFM_1_ intake, the conditions of harvest, postharvest, storage, and dairy feedstuffs production should be improved and regularly controlled in feedstuffs along the supply chain require prompt attention regarding AFB_1_ issues by veterinary competent authority.
